# The Electrical and Morphological Characteristics of Networks of Mechanically Exfoliated Nanosheets

**DOI:** 10.1002/smsc.202500417

**Published:** 2025-10-21

**Authors:** Luke Doolan, Yigit Sozen, Eoin Caffrey, Emmet Coleman, Tian Carey, Anthony Dawson, Cian Gabbett, Oran Cassidy, Jagdish K. Vij, Zdeněk Sofer, Andres Castellanos‐Gomez, Jonathan N. Coleman

**Affiliations:** ^1^ School of Physics CRANN & AMBER Research Centres Trinity College Dublin Dublin 2 Ireland; ^2^ 2D Foundry research group Instituto de Ciencia de Materiales de Madrid (ICMM‐CSIC) E‐28049 Madrid Spain; ^3^ Department of Electronic & Electrical Engineering Trinity College Dublin Dublin 2 Ireland; ^4^ Department of Inorganic Chemistry University of Chemistry and Technology Prague Technická 5 Prague 6 16628 Czech Republic

**Keywords:** nanosheet networks, printed electronics, semiconducting network

## Abstract

Solution‐processed nanosheet networks show great promise for the field of printed electronics due to their inherent scalability and competitive electrical properties. However, recent progress has allowed for the production of nanosheet networks by a dry, roll‐to‐roll mechanical exfoliation process. While this method is promising for producing low‐cost devices, the electrical properties of such networks are poorly understood and will require elucidation to enable optimization. Herein, the morphological and electrical properties of mechanically exfoliated networks of MoS_2_ are investigated. 3D images reveal that the networks show low porosity (11 ± 2%) and a high degree of in‐plane alignment. The network conductivity is dependent on annealing temperature and reaches a maximum of 11 ± 0.6 S m^−1^, when annealed at 300 °C. The networks show n‐type behavior with a mobility of 0.8 ± 0.1 cm^2^ V^−1^ s^−1^. Electrical impedance spectroscopy measurements reveal that this relatively low network mobility is caused by a combination of high inter‐nanosheet resistance (890 ± 150 kΩ) and low intrinsic mobility of the nanosheets (7 ± 2 cm^2^ V^−1^ s^−1^). Temperature‐dependent conductivity measurements show activated hopping as the internanosheet conduction mechanism near room temperature, with an activation energy of 61.9 ± 0.2 meV.

## Graphical abstract

MoS_2_ nanosheet networks made via dry exfoliation offer strong alignment, low porosity, and good conductivity. But charge mobility is held back by resistance at nanosheet junctions. Thinner sheets and better intersheet transport could boost performance, making these networks a strong contender for future printed electronics.

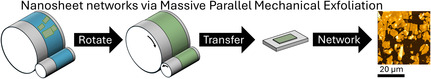

## 1 Introduction

Since the discovery of graphene monolayers produced by mechanical exfoliation, 2D materials have been an area of intensive research due to their exciting properties. While mechanical exfoliation is an excellent method to produce high‐quality nanosheets and investigate their properties, its industrial potential has been limited by its poor scalability.^[^ ^1^ ^]^ In comparison, exfoliation in liquids offers a cheap and scalable method to produce nanosheets.^[^ ^2^ ^]^ Such solution‐processed nanosheets can be deposited in a variety of different ways to form nanosheet networks:^[^ ^3^ ^]^ loosely connected assemblies of nanosheets, which are of interest in various fields including printed electronics.^[^ ^4^ ^]^ Nanosheet networks can be used to form a variety of electronic devices from transistors^[^ ^4^ ^]^ to sensors^[^ ^5^ ^]^ to capacitors,^[^ ^6^, ^7^ ^]^ with solution‐processed nanosheet networks displaying mobilities as high as ≈100 cm^2^ V^−1^ s^−1^.^[^ ^8^ ^]^ Although such solution‐processed networks show great promise, they do display some downsides. For example, the presence of organic residues associated with the solvent or stabilizers can result in unwanted doping or can hinder internanosheet charge transport if trapped within the junctions between nanosheets.^[^ ^3^, ^9^, ^10^, ^11^, ^12^, ^13^, ^14^, ^15^ ^]^

Recently, a highly‐scalable method to mechanically exfoliate nanosheets, dubbed massive parallel mechanical exfoliation (MPME), has been demonstrated.^[^ ^16^ ^]^ This method does not require solvent but utilizes rollers coated in adhesive tape to mechanically exfoliate a large number of nanosheets simultaneously. The flakes exfoliated by this method can be transferred onto a variety of substrates to form networks of conducting, semiconducting, or insulating nanosheets.^[^ ^16^ ^]^ Such networks can be stacked to form functional devices such as strain sensors and photodetectors.^[^ ^17^ ^]^ In principle, the lack of solvent and stabilizer means this method should be much cleaner than solution processing. However, contact with the tape will certainly leave behind some organic residues as has been shown by various groups.^[^ ^18^ ^]^ Nevertheless, compared to solution processing, mechanical exfoliation should result in relative clean networks with minimal residues as shown in ref. [19]

The electrical properties of nanosheet networks have improved drastically in recent years, with the mobility of solution‐processed MoS_2_ networks having increased from 0.1 cm^2^ V^−^ s^−1^ for liquid phase exfoliated (LPE) nanosheet networks measured in 2017, to ≈30–80 cm^2^ V^−1^ s^−1^ for highly optimized electrochemically exfoliated (EE) nanosheet networks today.^[^ ^20^, ^21^, ^22^ ^]^ In comparison, MPME MoS_2_ networks currently show relatively low mobilities of <2 cm^2^ V^−1^ s^−1^.^[^ ^16^ ^]^ However, if the electrical properties of MPME nanosheet networks could be improved, they could offer advantages over solution‐processed networks. To improve the performance of MPME networks, the factors limiting network conduction must first be understood. In general, the electrical properties of nanosheet networks depend on three factors: the properties of the nanosheets themselves, the morphology of the network and the nature of junctions between the nanosheets.^[^ ^3^ ^]^ To fully optimize the performance of these devices an understanding of each of these parameters, and the relationship between them, must be developed and quantified.

While the network mobility is affected by the nanosheet length and thickness, and to a lesser degree the carrier density, the main nanosheet‐related limiting factor is the nanosheet mobility.^[^ ^23^ ^]^ The intrinsic nanosheet mobility of high‐quality MoS_2_ is known to be phonon limited^[^ ^24^ ^]^ with additional contributions such as defect/impurity scattering^[^ ^25^ ^]^ further reducing it below its ultimate value. Although MoS_2_ nanosheets with mobilities as high as 150 cm^2^ V^−1^ s^−1^ have been reported,^[^ ^26^ ^]^ MoS_2_ nanosheets used in solution‐processed devices have tended to display mobilities <60 cm^2^ V^−1^ s^−1^.^[^ ^21^, ^23^, ^27^ ^]^ In addition, the nanosheet mobility is known to depend on the nanosheet thickness, with thicker nanosheets displaying lower mobility.^[^ ^28^, ^29^, ^30^, ^31^, ^32^ ^]^

Various morphological properties such as porosity and tortuosity are known to affect network conductivity and mobility.^[^ ^23^, ^33^ ^]^ Recent work has demonstrated that cross‐sectional microscopy can be used to determine a wide variety of morphological properties of nanosheet networks.^[^ ^8^, ^27^, ^33^, ^34^, ^35^, ^36^, ^37^, ^38^, ^39^ ^]^ The choice of imaging technique is important: while cross‐sectional transmission electron microscopy allows for visualization of the interface between overlapping nanosheets,^[^ ^8^, ^27^ ^]^ determination of larger‐scale properties—such as network porosity, pore shape and size, and nanosheet alignment—requires a large field of view (FOV). 3D imaging by focused ion beam‐scanning electron microscope nanotomography (FIB‐SEM nt) and nano X‐ray computed tomography (CT) offer resolution on the order of nanometers with FOVs on the order of 10 s of microns, which can be utilized to calculate morphological properties of nanosheet networks.^[^ ^33^, ^34^, ^38^ ^]^ In particular, FIB‐SEM nt, which offers improved resolution when compared to nano X‐ray CT, can be utilized to calculate a wide range of network properties from porosity to nanosheet alignment.^[^ ^33^, ^39^ ^]^

The reduced electrical performance of nanomaterial networks relative to their constituent nanomaterials has long been attributed to the resistance of the junctions between the individual particles in the network.^[^ ^40^, ^41^, ^42^, ^43^ ^]^ A number of methods have been proposed for the measurement of this junction resistance, from conductive atomic force microscopy (AFM)^[^ ^42^, ^44^ ^]^ to electrical impedance spectroscopy (EIS).^[^ ^45^ ^]^ We have recently derived a model which, when combined with EIS, can be used to calculate the nanosheet and junction resistances within a network.^[^ ^23^ ^]^ For example, this allows for direct measurement of junction and nanosheet resistances as a function of temperature^[^ ^23^ ^]^ or under stimuli such as strain.^[^ ^46^ ^]^ Measurement of the electrical properties of both nanosheets and the junctions between them offers insight for device optimization.

Here, we examine the morphological and electrical properties of MoS_2_ networks produced by MPME, showing that the networks are composed of well‐aligned nanosheets and show a low porosity. The electrical properties of the networks are limited both by the constituent nanosheets, which show a relatively low mobility, and by the junctions between the nanosheets. Temperature‐dependent electrical measurements reveal that conduction in the network is described by activated hopping between nanosheets.

## 2 Results and Discussion

### 2.1 Nanosheet Production and Characterization

**Figure 1:**
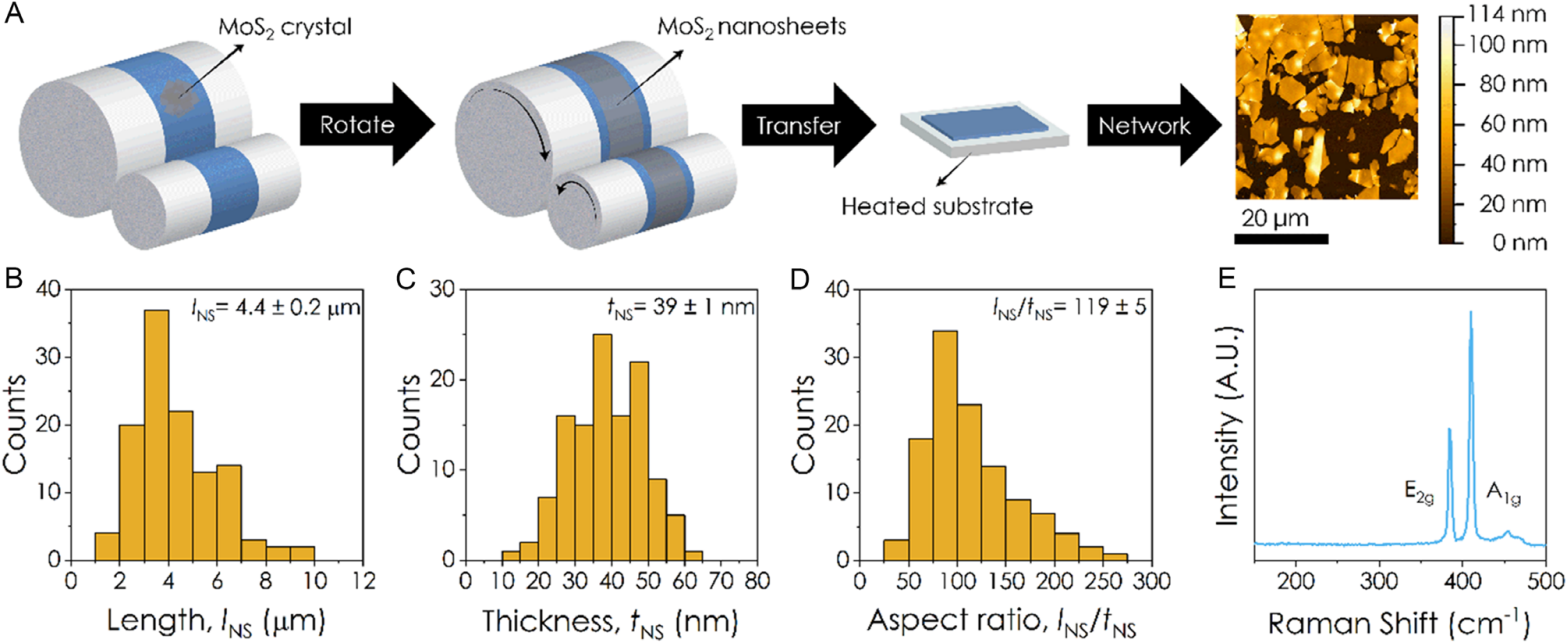
Nanosheet exfoliation and characterization. A) Schematic showing the process of MPME. MoS_2_ crystals are attached to tape on a large roller, the rotation of this roller causes the smaller roller to rotate, causing exfoliated nanosheets to be transferred to tape on the smaller roller. The exfoliated nanosheets are then transferred onto a substrate by thermal release process. Final image shows an AFM image of single transfer onto glass. B–D) Distribution of nanosheet length (defined as longest axis), thickness and aspect ratio (length divided by thickness) for MoS_2_ nanosheets exfoliated by MPME, based on AFM measurements. The average nanosheet length is *l*_NS_ = 4.4 ± 0.2 μm, the average nanosheet thickness is *t*_NS_ = 39 ± 1 nm while the average nanosheet aspect ratio is *l*_NS_/*t*_NS_ = 119 ± 5. E) Raman spectrum of a MPME MoS_2_ nanosheets showing the characteristic E_2g_ and A_1g_ peaks of 2H‐MoS_2_. For the histograms in (B), (C), and (D), the sample size was *n* = 119.

MoS_2_ nanosheets were exfoliated using MPME, a process which is shown schematically in **Figure** **1**A and utilizes two rollers of different sizes in contact with each other. Nitto SPV 224 tape is attached to each of the rollers and crystal MoS_2_ is attached to tape on one of the rollers. The rollers are placed in contact and are rotated using a drill, leading to the repeated mechanical exfoliation of the MoS_2_: after this process both pieces of tape are coated in MoS_2_ nanosheets.^[^ ^16^ ^]^ The nanosheets are then transferred to a substrate for characterization, the thickness of the network can be controlled by repeated deposition, with a single deposition creating a sparse network which allows for characterization of individual exfoliated nanosheets, while multiple depositions create thick homogenous networks which can be used for a variety of applications.^[^ ^17^ ^]^ Natural MoS_2_ crystal was used in this process to make samples that were comparable to previously reported EE MoS_2_ networks.^[^ ^22^, ^23^ ^]^

The dimensions of the MPME nanosheets were measured from AFM images of a sparse network obtained by a single deposition. A representative AFM image is shown in Figure 1A (right). Using these images, the length, width, and thickness of 119 nanosheets was measured. The distribution of nanosheet length (*l*_NS_, i.e., the longest dimension) is shown in Figure 1B and yields a mean length of 4.4 ± 0.2 μm. We found the nanosheet width, *w*_NS_, to be close to *l*_NS_, such that on average *l*_NS_~1.4*w*_NS_ (see Supporting Information). The nanosheet thickness distribution is shown in Figure 1C and appears to be normally distributed, with an average thickness of 39 ± 1 nm. Although these MPME nanosheets are considerably larger than reports for solution‐processed transition metal dichalcogenide (TMD) nanosheets,^[^ ^47^ ^]^ their large thickness results in mediocre aspect ratios. We also plot the nanosheet aspect ratio (*l*_NS_/*t*_NS_) distribution (Figure 1D), finding a mean value of 119 ± 5. This value is above the maximum achievable average aspect ratio for LPE TMD nanosheets (≈30),^[^ ^47^ ^]^ however, it is low compared to the average aspect ratio of EE TMD nanosheets which range between 100–2000.^[^ ^22^, ^48^, ^49^, ^50^, ^51^ ^]^

Raman spectroscopy was carried out to characterize the quality of the exfoliated nanosheets. The Raman spectrum can be seen in Figure 1E, showing the characteristic A_1g_ and E_2g_ peaks of the 2H phase of MoS_2_,^[^ ^52^ ^]^ with no evidence of the J_2_ and the J_3_ peaks of the metallic 1T phase, located between 200 and 350 cm^−1^,^[^ ^53^ ^]^ confirming that the MoS_2_ is in the semiconducting 2H phase. In addition, as with EE MoS_2_ nanosheets,^[^ ^49^ ^]^ we see no sign of the longitudinal acoustic mode at ≈220 cm^−1^,^[^ ^54^ ^]^ which becomes activated in the presence of lattice defects, indicating our nanosheets to be relatively high‐quality. According to ref. [55] the absence of this peak implies a basal plane defect density of <0.05 nm^−2^.

### 2.2 Network Morphology

**Figure 2:**
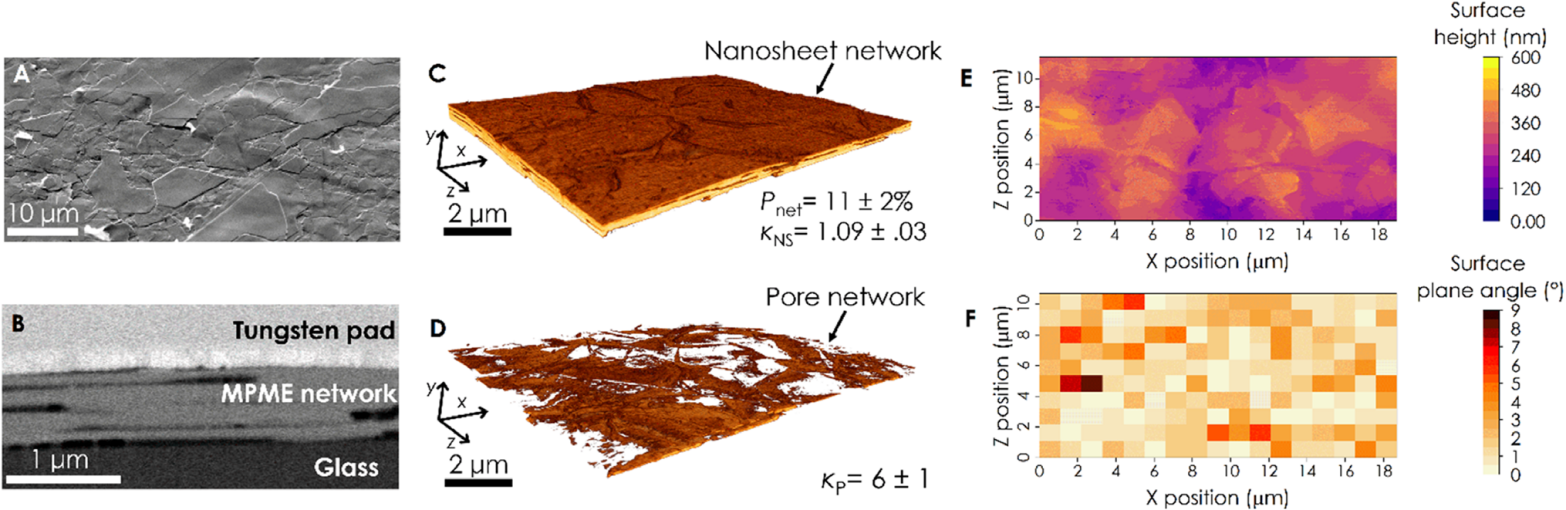
Network morphological characterization. A) SEM image of the surface of a MPME MoS_2_ network. B) SEM cross‐sectional image showing the internal structure of the network shown in (A). C,D) FIB‐SEM nt 3D image of the nanosheet and pore network, respectively. 3D images were generated from stacks of successive SEM cross‐sections (*n* = 678). From the images an average network porosity of 11 ± 2% was calculated. The in‐plane tortuosity factor of the nanosheet and pore volume was also calculated as *κ*_NS_ = 1.09 ± .03 and *κ*_P_= 6 ± 1, respectively. E) Surface height map of the network calculated from (C). F) Angle of planes fit to the surface shown in (E). The planes had a size of 1100 × 1100 nm, ¼ of *l*_NS_. The surface has an average angle of 1.7 ± 0.1º (*n* = 170).

While a single transfer allows for calculation of the properties of the exfoliated nanosheets, the electrical properties of nanosheet networks produced by a single transfer are limited due to incomplete coverage. Repeated depositions can be used to increase the network thickness resulting in an increases in mobility and conductivity, due to percolation effects, with conductivity of MPME graphene networks saturating at 6 × 10^4^ S m^−1^ for five depositions.^[^ ^16^, ^17^ ^]^ To produce MoS_2_ films which show bulk conductivity, thicker networks were produced by repeated transfers onto the same area. For example, we found six transfers yields a network with mean thickness of 281 ± 10 nm. The surface morphology of a six transfer MPME MoS_2_ network is shown in **Figure** **2**A. This SEM image shows a network of well‐aligned nanosheets and appears much more similar to networks of high aspect ratio EE nanosheets than those of low‐aspect ratio LPE nanosheets.^[^ ^21^, ^22^, ^23^, ^39^, ^56^, ^57^ ^]^

A cross‐sectional FIB‐SEM image of the network can be seen in Figure 2B, allowing for the internal structure of the network to be examined. The network is composed of well‐aligned nanosheets, some of which appear to have large overlap areas, while there is evidence of long slit‐like pores between some of the nanosheets. The quantitative information that can be obtained from a single cross‐section is limited. This is unfortunate as the morphology of a network largely controls its electrical properties and so determines performance in a wide variety of applications from transistors to sensors.^[^ ^4^, ^58^ ^]^ As mentioned in the introduction, FIB‐SEM nt is an effective tool to quantitatively interrogate the morphology of networks of nanomaterials.^[^ ^33^ ^]^ This technique uses an automated procedure to repeatedly slice and image cross‐sectional surfaces. The individual cross‐sections can then be stitched together to form a 3D image. Here, FIB‐SEM nt was carried out to understand the internal structure of the MPME MoS_2_ networks. The in‐plane resolution of the images is 5 nm and the distance between the slices is ≈15 nm, the 3D image contains ≈700 million voxels which contain a large amount of information about the network. To quantitatively understand the network morphology, the images must be binarized into their components. Four‐way segmentation was carried out using trainable WEKA segmentation,^[^ ^59^ ^]^ with the protective top tungsten pad, the MoS_2_ nanosheets, the pores, and the glass substrate all separated. This allows for the generation of 3D images of both the nanosheet and pore network, a section of which can be seen in Figure 2C,D, showing a well‐connected network of MoS_2_ nanosheets with low surface roughness and a pore network that is composed of large slit‐like pore chambers.

From the binarized images, a wide variety of network properties can be calculated. Firstly, the visible network porosity, *P*_Net_, can be calculated, yielding a value of 11 ± 2%. This value is considerably lower than the values that have been reported for LPE TMD nanosheet networks (40%–60%) but is above the value of 2%–5% measured for EE TMD nanosheets networks.^[^ ^33^, ^49^ ^]^ Another important parameter for network electronics is the nanosheet network tortuosity factor. The tortuosity factor describes the reduction in conductivity of a network due to convolution of the conduction path.^[^ ^60^ ^]^ The nanosheet network shows a small in‐plane tortuosity factor of *κ*_NS_ = 1.09 ± 0.03, just above the minimum value of 1 suggesting charge transport through the network is minimally impeded by geometric constraints.

Interestingly, despite the network's relatively low porosity, the pore volume forms a well‐connected network with an in‐plane pore tortuosity factor of *κ*_P_ = 6 ± 1, this suggests that while it is possible to travel from one side of the pore network to the other, travel will be impeded by long, convoluted paths. To further understand the pore network, the connectivity of the network was calculated. This parameter describes the percentage of the porosity contained in the largest connected pore volume. The pore network has a connectivity value of ≈90% confirming that the pore volume is highly connected, if quite tortuous. Due to the connectivity of the pore network, analysis of the shape of individual pores is difficult as it requires artificially separating individual pore chambers. Here, we use the area‐weighted pore fraction^[^ ^33^ ^]^ to understand which pores contribute significantly to the overall porosity (see Supporting Information) and use the resulting definition to calculate the average pore cross‐sectional area (40 000 ± 1,000 nm^2^), which corresponds to an average characteristic pore length (square root of pore area) of 151 ± 8 nm. The pores show an average circularity of 0.19 ± 0.01, consistent with slit‐like pores (the circularity is a measure of how circular the pores are, with a value of 1 suggesting a perfect circle and values below 1 describing a tendency toward higher aspect ratio pores). The calculated pore length is below the values reported for spray‐coated LPE graphene networks. However, the pore circularity is similar to networks composed of large LPE graphene nanosheets but is below the value of networks composed of small nanosheets.^[^ ^33^ ^]^ This demonstrates that the network is composed of small slit‐like pores, although the limited network thickness may mean the pore morphology is not fully developed.

Figure 2C suggests the network has a relatively smooth surface, which is important for forming electronic devices, where stacking a variety of materials is required. From the 3D image, the height of the network can be calculated locally on a pixel‐by‐pixel basis. A heat map of the surface height can be seen in Figure 2E. The surface roughness of the network can be calculated from the map yielding a root mean square value of *S*_q_ = 59 ± 2 nm for this network which has a mean thickness of 281 ± 10 nm, equivalent to a fractional roughness of 21 ± 1.4%. This value is most probably correlated with the nanosheet thickness. The fractional value is above that reported for very thick spray‐coated LPE graphene networks (126 nm for a 1.231 μm network, 10%) but similar to the value calculated for spray‐coated EE graphene networks (24 nm for a 123 nm network, 20%) by the same method.^[^ ^39^ ^]^ The roughness of solution‐processed networks is strongly dependent on the deposition technique, for LPE graphene networks, roughness values of 9 and 32 nm have been reported for transfer and screen printed networks, although in these cases, the thickness was not reported.^[^ ^61^, ^62^ ^]^

Nanosheet alignment is an important parameter for network electronics as good alignment leads to large internanosheet overlap areas, yielding improved electrical contact between the nanosheets and so reduced junction resistance.^[^ ^3^ ^]^ To calculate the nanosheet alignment from 3D images, the network surface can be divided into a grid of equally sized tiles. Each surface tile is then approximated as a 2D plane using least squares fitting and the polar angle between its normal vector and *Y*‐axis is used to determine the nanosheet alignment at the surface of the network (see Supporting Information). A tile with a surface angle of 0° has a normal vector that is parallel to the *Y*‐axis, meaning it is perfectly flat, or aligned in the plane of the film. A heat map of the measured surface plane angles is shown in Figure 2F using a plane size of 1100 × 1100 nm (1/4 of *l*_NS_, see Supporting Information). Using this method an average surface angle of 1.7 ± 0.1° was calculated. This value is below the values calculated for spray‐coated EE (3.16°) and LPE (15.2°) graphene networks.^[^ ^39^ ^]^ This high degree of in‐plane alignment, combined with the low porosity and nanosheet tortuosity factor suggests that the network should show excellent electrical properties.

### 2.3 Network Electrical Properties

**Figure 3:**
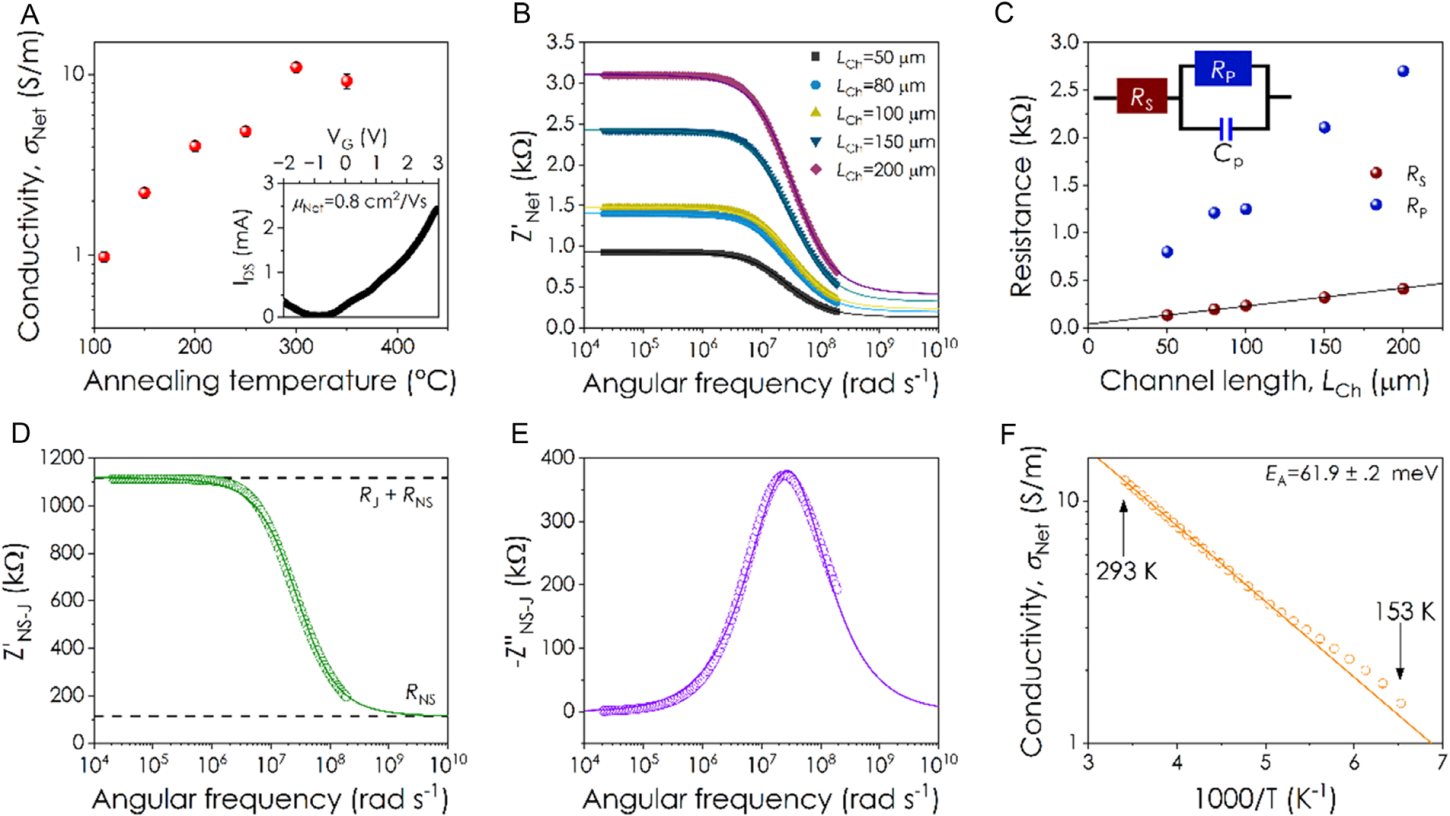
Network electrical properties. A) Conductivity of a MPME MoS_2_ network as a function of annealing temperature. The network was annealed for 2 h under vacuum at each temperature except for 110 ºC, which is the network as it was deposited. Inset: Transfer curve for an ionically gated mechanically exfoliated MoS_2_ network, yielding a network of mobility of 0.8 ± 0.1 cm^2^ V^−1^ s^−1^ and an on/off ratio of 80. Measured with a source‐drain voltage of 1 V. B) Real component of the impedance spectrum, of a MPME MoS_2_ network, as a function of the angular frequency of the applied voltage. Spectrum is shown for five channel lengths with constant channel width. C) Series (*R*_s_) and parallel (*R*_p_) resistances as a function of channel length. The real component of the impedance spectra, shown in (C), were fit using a model containing a resistor in series with a resistor in parallel with a capacitor, circuit diagram shown as an inset. The series resistance varies linearly with channel length and allows for calculation of the contact resistance. D) Real component of the impedance spectrum of a NS‐J pair in a MPME MoS_2_ network. Using this spectrum, the nanosheet and junction resistance and the junction capacitance can be calculated. E) Imaginary component of the impedance spectrum of a NS‐J pair in a MPME MoS_2_ network. Fitting allows for the calculation of the junction resistance and capacitance. F) Network conductivity as a function of inverse temperature. The straight line is a fit to equation 3 and is consistent with an activation energy of 61.9 ± 0.2 meV. The data in this figure comes from a single sample. However, preliminary experiments on multiple samples showed these results to be reproducible and reasonably consistent. The error bars in (A) are associated with uncertainty in film thickness while those in (C) (too small to see) are fitting errors.

To measure the electrical properties of the networks, interdigitated electrodes (IDEs) with varying channel lengths were patterned onto the sample. Initially, the network conductivity, *σ*_Net_, was 0.98 ± 0.07 S m^−1^, this value is considerably higher than what has been achieved with LPE MoS_2_ networks (10^−6^–10^−3^ S m^−1^),^[^ ^23^, ^63^ ^]^ but is below the state of the art for EE MoS_2_ networks (10^−1^–10^3^ S m^−1^).^[^ ^22^, ^23^, ^36^, ^51^, ^57^ ^]^ It has previously been found that annealing MPME MoS_2_ networks at 150 °C overnight under vacuum led to a doubling in network mobility.^[^ ^16^ ^]^ To investigate the effect of annealing on the electrical conductivity, the networks were annealed under vacuum for 2 h at increasing temperature. As shown in **Figure** **3**A, the conductivity increased with annealing temperature up to 300 °C, with a maximum conductivity of 11.0 ± 0.7 S m^−1^ achieved. This increase in conductivity is similar to behavior observed for EE MoS_2_ networks, which have been found to show a mobility maximum after annealing at 250 °C and a decrease thereafter.^[^ ^20^ ^]^ It is still not completely clear what is the mechanism of conductivity enhancement by annealing in nanosheet networks. However, it is almost certainly due to removal of adsorbates within junctions. While we cannot rule out changes in the quality of junction interfaces due to nanosheet reconfiguration, such effects seem unlikely at the relatively low temperatures used here.

In order to further understand the networks electrical properties we used gating measurement to measure the mobility of an annealed network, *μ*_Net_. Due to the thickness of the networks (≈250 nm), dielectric gating of the entire channel volume was not possible, and so the networks were gated using an ionic liquid (1‐ethyl‐3‐methylimidazolium bis(trifluoromethylsulfonyl)imide (EMIM‐TFSI)). The gate voltage was swept from −3 to 3 V, with a source‐drain voltage of 1 V. A representative transfer curve can be seen in Figure 3A (inset) and shows predominantly n‐type behavior, as is generally found for networks of MoS_2_,^[^ ^21^, ^27^, ^49^ ^]^ with an on:off ratio of ≈100. To calculate the mobility the volumetric network capacitance (*C*_V_) was calculated by taking the value from Carey et al.^[^ ^48^ ^]^ and adjusting it to account for the nanosheet thickness in accordance with the equation relating *C*_V_ to nanosheet thickness in networks of 2D materials,^[^ ^64^ ^]^ yielding a value of 0.45 F cm^−3^ (see Supporting Information for more detail). Taking the slope of the *I*_DS_ versus *V*_G_ curve in the range 2 V < V_G_ < 3 V yields an average network mobility, *μ*_Net_, of 0.8 ± 0.1 cm^2^ V^−1^ s^−1^.This is in line with previously calculated values for MPME MoS_2_ networks measured by dielectric gating (0.03–1.36 cm^2^ V^−1^ s^−1^ for single deposition devices with relatively short channel lengths of 10 μm). Our value is larger than those reported for LPE MoS_2_ printed networks (0.001–0.1 cm^2^ V^−1^ s^−1^)^[^ ^21^, ^65^ ^]^ but below values in the literature for EE MoS_2_ printed transistors (0.2–80 cm^2^ V^−1^ s^−1^).^[^ ^20^, ^22^, ^23^, ^49^, ^57^, ^66^, ^67^, ^68^ ^]^ A summary of the morphological and electrical properties of MPME, LPE, and EE MoS_2_ networks can be found in **Table** **1**.

**Table 1:** Summary of properties of MoS_2_ networks produced by MPME (this work), EE, and LPE.

	MPME [this work]	LPE	EE
Nanosheet aspect ratio	119 ± 5	≈30^[^ ^47^ ^]^	100–2000^[^ ^22^, ^48^, ^49^, ^50^, ^51^ ^]^
Network porosity	11 ± 2%.	40%–60%^[^ ^33^ ^]^	2%–5%^[^ ^49^ ^]^
Network conductivity	11.0 ± 0.7 S m^−1^	10^−6^–10^−3^ S m^−1^^[^ ^21^, ^63^ ^]^	10^−1^–10^3^ S m^−1^^[^ ^22^, ^23^, ^36^, ^49^, ^51^, ^57^ ^]^
Network mobility	0.8 ± 0.1 cm^2^ V^−1^ s^−1^	10^−3^–10^−1^ cm^2^ V^−1^ s^−1^^[^ ^21^, ^65^ ^]^	0.2–80 cm^2^ V^−1^ s^−1^^[^ ^20^, ^22^, ^23^, ^49^, ^57^, ^66^, ^67^, ^68^ ^]^
Nanosheet resistance	120 ± 20 kΩ	4–192 MΩa) ^[^ ^23^ ^]^	100–670 kΩ^[^ ^23^, ^46^, ^51^ ^]^
Junction resistance	890 ± 150 kΩ	6–24 GΩa) ^[^ ^23^ ^]^	350–2,900 kΩ^[^ ^23^, ^46^, ^51^ ^]^
Nanosheet mobility	7 ± 2 cm^2^ V^−1^ s^−1^	47 cm^2^ V^−1^ s^−1^	37–42 cm^2^ V^−1^ s^−1^^[^ ^23^, ^51^ ^]^
*R*_J_/*R*_NS_	7 ± 2	125–1400^[^ ^23^ ^]^	3.1–5.3^[^ ^23^, ^46^, ^51^ ^]^
Junction area	2.6 μm^2^	Point like [≈100 nm^2^]	0.4 μm^2^^[^ ^23^, ^46^ ^]^
Activation energy	61.9 ± 0.2 meV	260–520 meV^[^ ^3^, ^21^, ^65^, ^74^ ^]^	28–96 meV^[^ ^23^, ^36^, ^57^ ^]^

a) Values are for WSe_2_ and WS_2_ networks.

Combined with the network conductivity after annealing reported above, this network mobility implies a network carrier density of *n*_Net,_ = (8.6 ± 1.1) ×10^23^ m^−3^, consistent with reports for EE MoS_2_ networks produced from MoS_2_ crystal, where values in the range 10^22^–10^25^ m^−3^ have been calculated.^[^ ^22^, ^23^, ^36^, ^51^, ^57^, ^68^ ^]^ The network carrier density can be related to the nanosheet carrier density, *n*_NS,_ by *n*_net_ = *n*_NS_(1–*P*_Net_),^[^ ^21^ ^]^ yielding a nanosheet carrier density of (9.6 ± 1.3) × 10^23^ m^−3^. This value is in line with previous data for the carrier density of natural MoS_2_ crystals, which span 10^21^–10^24^ m^−3^, with the broad range probably attributable to impurities.^[^ ^69^, ^70^ ^]^ This suggests that the high carrier density of EE MoS_2_ networks is likely to be associated with the starting material and not the liquid exfoliation process.

The measured network mobility is well below previously reported values for the intrinsic mobility of MoS_2_ (which we refer to as *μ*_NS_).^[^ ^29^, ^30^, ^71^ ^]^ However, nanosheet networks always display *μ*_net_ < *μ*_NS_, due to the limiting effects of internanosheet junctions.^[^ ^3^ ^]^ Recently, we reported a model which can be used to show that the mobility of a nanosheet network with reasonably high carrier density and low nanosheet tortuosity factor can be described by^[^ ^23^ ^]^

(1) $$\mu_{\text{Net}}\approx \frac{\mu_{\text{NS}}}{1+R_{J}/R_{\text{NS}}}$$

where *R*_NS_ is the mean nanosheet resistance and *R*_J_ is the mean resistance of the junctions between the nanosheets. Thus, it is clear from this equation that our relatively low value of *μ*_net_ is simply a manifestation of the fact that *R*_J_ > *R*_NS_.

It is not always clear whether the fact that *R*_J_ > *R*_NS_ is predominately due to high values of *R*_J_ or relatively low values of *R*_NS_. The nanosheet resistance is given by *R*_NS_ = (2σ_NS_ *t*_NS_)^−1^,^[^ ^23^ ^]^ where σ_NS_ and *t*_NS_ are the nanosheet conductivity and thickness, respectively. Here, the nanosheet thickness of *t*_NS_ = 39 ± 1 nm is considerably higher than values reported in the literature for EE MoS_2_ (0.6–15 nm),^[^ ^20^, ^22^, ^27^, ^36^, ^37^ ^]^ and is somewhat higher than typical LPE MoS_2_ nanosheets (>25 nm).^[^ ^21^ ^]^ This causes our MPME nanosheets to have low *R*_NS_ values, resulting in a corresponding increases in *R*_J_/*R*_NS_. However, our *μ*_Net_ values are higher than those for LPE networks. Combined with the expectation that MPME nanosheets have lower *R*_NS_ compared to LPE nanosheets, this suggests that MPME networks have junction resistances which are lower than those of LPE nanosheet networks (where junction resistances as high as 24 GΩ have been measured^[^ ^23^ ^]^). However, to fully understand the factors limiting *μ*_Net_ in these, or any networks, it is necessary to measure both *R*_J_ and *R*_NS_.

It has recently been shown that both *R*_NS_ and *R*_J_ can be measured by EIS.^[^ ^23^ ^]^ EIS is a technique which analyses how an induced AC current depends on the frequency of an applied AC voltage to infer material properties. Here, we measured impedance spectra of an MPME MoS_2_ network at five different channel lengths, *L*_Ch_, with the resultant real impedance ($Z\prime_{Net}$) spectra shown in Figure 3B. The shape of the nanosheet network impedance spectra implies that they can be fit using a model representing an equivalent circuit composed of a resistor (*R*_S_) in series with a parallel combination of a resistor (*R*_P_) and a capacitor (*C*_P_)—a schematic of the equivalent circuit is shown as an inset in Figure 3C. Here, the value of *R*_s_ represents the portion of the total network resistance associated with the nanosheets plus the contact resistance at the electrode‐network interface (which is in series with the combined nanosheet resistance).^[^ ^23^ ^]^ In addition, *R*_P_ is the portion of the total network resistance associated with the junctions while *C*_P_ is the capacitance of the network which is associated with the array of junctions within the network (rather than the intrinsic MoS_2_ permittivity).^[^ ^23^ ^]^ We note that, because of the distribution of junction resistances and capacitances within the network, better agreement between model and data can be achieved by replacing the capacitor with a constant phase element.^[^ ^72^ ^]^ The resultant fitting equations are given in the Supporting Information.

Fits obtained using this model are plotted as solid lines in Figure 3B and show good agreement with the data. The resultant values of *R*_S_ and *R*_P_ as shown in Figure 3C. Both vary linearly with channel length, as the number of both nanosheets and junctions within the channel scale with channel length. However, we expect the *R*_S_ versus *L*_Ch_ to have a positive intercept associated with the contact resistance at the electrode/channel interface. Fitting this data yields a value for 2*R*_c_ of 38 ± 5 Ω, a value which is small relative to both the *R*_S_ values and the total channel resistance, indicating that these networks are not contact‐limited. This value corresponds to a specific contact resistance of 370 ± 20 kΩ μm at each contact. This value is in line with, although at the upper end of, previously calculated contact resistances of EE MoS_2_ nanosheet networks with gold contacts (100–400 kΩ μm).^[^ ^22^, ^23^, ^67^ ^]^ The high value calculated here may be due to the presence of a 3 nm Ti layer between the network and the Au, which was used to improve the adhesion of the contacts to the substrate. Ti is known to show a higher contact resistance than Au with MoS_2_.^[^ ^73^ ^]^ We note that EIS is an excellent way to calculate the contact resistance as variations in the junction resistance across the sample can lead to significant scatter in total resistance versus channel length data, leading to inaccurate calculated *R*_c_ values (see Supporting Information). Moreover, accurate knowledge of the contact resistance allows us to contact‐resistance‐correct $Z\prime_{Net}$ by subtracting 2*R*_c_ (the imaginary impedance is not affected by series resistance and so does not need correction).

The model underpinning Equation (1) can be written in a way that allows one to convert from network impedance (after *R*_c_‐correction) to the impedance of a single, average nanosheet‐junction (NS‐J) pair within the network^[^ ^23^ ^]^

(2) $$Z_{\text{NS}-J}=Z_{\text{Net}}\frac{A_{\text{Net}}}{L_{\text{Ch}}}\frac{\left(1-P_{\text{Net}}\right)}{2t_{\text{NS}}}$$

where *Z*_NS‐J_ and *Z*_Net_ are the impedance of an average NS‐J pair and the network, respectively, and *A*_Net_ is the network cross‐sectional area (channel width × network thickness). Using this equation allows one to obtain the impedance spectra associated with a single (average) NS‐J pair. The resultant real and imaginary spectra associated with an NS‐J pair is shown in Figure 3D,E. Microscopically, we expect such a NS‐J pair to resemble a resistor representing the nanosheet (*R*_NS_) in series with a parallel resistor/capacitor combination (*R*_J_, *C*_J_) representing the junction (similar to the equivalent circuit shown in the inset of Figure 3C). This allows us a to fit the data in Figure 3D,E (see Supporting Information for equations) to obtain the nanosheet and junction resistance as well as the junction capacitance.^[^ ^23^ ^]^ Fitting these spectra yields values of *R*_NS_ = 120 ± 20 kΩ, *R*_J_ = 890 ± 150 kΩ, and *C*_J_ = 35 ± 5 fF, as well as a parameter describing the width of the *R*_J_ and *C*_J_ distributions:^[^ ^72^ ^]^ *n *= 0.819 ± 0.003 (n = 1 for infinitesimally narrow distributions).

The nanosheet resistance is well below the value recently obtained for LPE TMD nanosheets (4–192 MΩ), but is consistent with the values found for EE MoS_2_ nanosheets (100–670 kΩ).^[^ ^23^, ^46^, ^51^ ^]^ Combining the nanosheet resistance with the measuring nanosheet thickness, the nanosheet conductivity can be calculated, yielding a value of σ_NS_ = 110 ± 20 S m^−1^. Taking the calculated nanosheet carrier concentration of n_NS_ = (9.6 ± 1.3) × 10^23^ m^−3^ yields a nanosheet mobility *μ*_NS_ = 7 ± 2 cm^2^ V^−1^ s^−1^ (the same value is obtained if we use Equation 1 to combine the measured network mobility with the values of *R*_NS_ and *R*_J_ measured by impedance). This value is considerably below that generally reported for thin nanosheets of MoS_2_ (*μ*_NS_~40–200 cm^2^ V^−1^ s^−1^).^[^ ^29^, ^30^, ^31^, ^71^ ^]^ However, our MPME flakes are very thick and it is well known that mobility in MoS_2_ falls off with increasing nanosheet thickness.^[^ ^28^, ^29^, ^30^, ^31^ ^]^ Indeed, one article found that, while five layer MoS_2_ nanosheets had mobility of ≈100 cm^2^ V^−1^ s^−1^, a 25 layer thick nanosheet showed a mobility of <10 cm^2^ V^−1^ s^−1^.^[^ ^28^ ^]^ It is worth noting that, when we use a model such as the one underpinning Equation 1,^[^ ^23^ ^]^ the parameters (e.g., *μ*_NS_) tend to represent average or effective values, rather than intrinsic values. For example, factors such as nanosheet thickness, defect content or charge trapping can reduce the value of *μ*_NS_ associated with the nanosheets within the network under study relative to intrinsic values for isolated nanosheets. Then, analysing data using the model will return a value of *μ*_NS_ which incorporates the effect of these factors.

As described in ref. [23] the junction capacitance can be modeled as a simple parallel plate capacitor $C_{J}=\varepsilon_{0}\varepsilon_{r}A_{J}/d$, where *A*_J_ is the junction area and *d* is the nanosheet separation. Although ref. [19] shows mechanically exfoliated MoS_2_ to be quite clean, some residue is expected.^[^ ^18^ ^]^ Thus, we approximate the separation as *d*~1 nm, and assume ε_r_ = 1.5, intermediate between vacuum and organic hydrocarbons (to simulate a partial coating). This suggests an overlap area between the nanosheets of ≈2.6 μm^2^, considerably larger than the value of ≈0.4 μm^2^ obtained for EE MoS_2_,^[^ ^23^, ^46^ ^]^ demonstrating that the larger nanosheets produced by MPME produce large area junctions. However, we note that mean nanosheet lateral dimensions imply a mean nanosheet area of ≈11 μm^2^ (approximating them as ellipses) This means the average junction area is 24% of the average nanosheet area, slightly below values of 33%–40% found for EE MoS_2_ networks.^[^ ^23^, ^46^ ^]^ This may imply that, because the thicker MPME nanosheets are less flexible than EE nanosheets, therefore, they are less able to generate junctions where the nanosheets conform perfectly to each other, resulting in smaller fractional junction areas.

The calculated value of *R*_J_ (890 ± 150 kΩ) is well below values previously measured for LPE TMD nanosheets (6–24 GΩ) and is in the range of values measured previously for EE MoS_2_ nanosheets (350–2,900 kΩ).^[^ ^23^, ^46^, ^51^ ^]^ However, it is more appropriate to compare values for the product of junction resistance and junction area, (*RA*)_J_. Here, we find (*RA*)_J_ ≈ 2.3 × 10^−6^Ω m^2^, somewhat larger than the values of (0.15–1.2) ×10^−6^Ω m^2^ associated with EE MoS_2_.^[^ ^23^, ^46^ ^]^ The higher (*RA*)_J_ values found here clearly indicate that the thicker MPME nanosheets give junctions that are inferior to those obtained with EE nanosheets, even though MPME junctions should be cleaner. This may be because they do not create quite as conformal junctions as thinner nanosheets.

The ratio of nanosheet to junction resistance (*R*_J_/*R*_NS_) can be used to determine what limits charge transport in a nanosheet network. Here, we find a value of 7 ± 2, which suggests that the networks are junction‐limited but not excessively so. This value is lower than has been calculated for LPE TMD networks (125–1400) but is above the values calculated for EE MoS_2_ nanosheet networks (3–5).^[^ ^23^, ^46^, ^51^ ^]^

Further insight into charge transport in the network can be obtained by investigating the temperature‐dependent DC conductivity, as shown in Figure 3F. The conductivity increases with increasing temperature as is generally found for nanosheet networks, where the conduction is described by hopping of charge carriers across junctions.^[^ ^23^, ^74^ ^]^ Because *R*_J_/*R*_NS_ is significantly above 1, we can safely treat the conductivity here as junction‐limited. Activation energy analysis (see Supporting Information) reveals that the network conduction is described by activated hopping near room temperature, which is described by

(3) $$\sigma (T)=\sigma_{0}\exp\left(-\frac{E_{a}}{k_{B}T}\right)$$

where *σ*_0_ is a constant and *E*_*a*_ is the activation energy required to hop across the junction. Fitting the data with this equation yields *E*_*a*_ = 61.9 ± 0.2 meV. This value is well below the values of 260–520 meV reported for LPE MoS_2_ networks.^[^ ^3^, ^21^, ^65^, ^74^ ^]^ This value is however similar to the values measured for EE MoS_2_ networks which range between 28–96 meV.^[^ ^23^, ^36^, ^57^ ^]^ Thus, it is clear that the intersheet charge transport in the MPME networks is much more similar to that in networks of EE nanosheets with large area junctions compared to networks of LPE nanosheets which display point‐like junctions. In the future, it will be important to elucidate the relationship between junction geometry and activation energy.

Based on our results, we can suggest methods for optimizing the electrical properties of semiconducting MPME networks. We believe the mobility of the MPME MoS_2_ nanosheet networks is predominately limited by the nanosheet thickness, such that reducing nanosheet thickness would increase *μ*_Net_. Firstly, as described above, thinner nanosheets have higher intrinsic mobility. Secondly decreasing the nanosheet thickness would increase the nanosheet resistance and thus decrease *R*_J_/*R*_NS_. Thirdly, decreasing nanosheet thickness would make the nanosheets more flexible, this allowing the formation of more conformal junctions. This would decrease (*RA*)_J_ and so *R*_J_. However, it is not yet clear, how the MPME method can be modified to allow control of nanosheet thickness.

## 3 Conclusion

In conclusion, we have investigated the morphological and electrical properties of networks of MoS_2_ produced by MPME. The exfoliation yields large, thick, high aspect ratio nanosheets. Networks can be produced by repeat deposition of these nanosheets. The networks show a low porosity of 11 ± 2%, a high degree of nanosheet alignment, making them an ideal candidate for electronic devices. The networks show increasing conductivity with annealing, up to a temperature of 300 °C, however, they show relatively low mobilities of 0.8 ± 0.1 cm^2^ V^−1^ s^−1^. Using EIS, the nanosheet and junction resistance were calculated for the networks. This yielded a nanosheet conductivity of 110 ± 20 S m^−1^ and a junction resistance of 890 ± 130 kΩ. The calculated nanosheet resistance suggests that the nanosheets show a low mobility of 7 ± 2 cm^2^ V^−1^ s^−1^, which is likely caused by the thickness of the nanosheets and their high carrier density. Conduction in the networks is described by activated hopping, with an activation energy of 61.9 ± 0.2 meV.

## 4 Experimental Section

4.1

4.1.1

##### MPME

The nanosheet films of van der Waals materials were produced with high‐throughput mechanical exfoliation, which is an automated exfoliation method based on in‐contact rolling of the two parallel cylinders. First, Nitto SPV 224 tapes were placed onto cylinders via previously attached double‐sided tapes, with their adhesive side facing outward. The bulk crystals of a 2D material to be exfoliated was uniformly transferred onto one of the Nitto tapes, over an area of 3 cm^2^. The cylinders were rolled in contact for 20 s using a drill motor to obtain a large‐area nanosheet film formation (≈13 cm^2^) on the acceptor Nitto tape. For the transfer of films, we adhered the Nitto tape onto a glass substrate and annealed it at 110 °C for 5 min. Several transfers can be performed to obtain a homogeneous and continuous film coverage on the substrate.

##### Raman Spectroscopy

A Renishaw Raman spectrometer, utilizing a 532 nm laser and equipped with a 100× objective, was employed to collect spectra. To minimize potential thermal damage, the incident laser power was kept at ≈1 mW.

##### AFM

As‐deposited samples on glass were used for AFM analysis. A Bruker Multimode 8 scanning probe microscope was operated in ScanAsyst mode (noncontact) under ambient conditions, using aluminum‐coated silicon cantilevers (OLTESPA‐R3). 119 nanosheets were measured to obtain statistical data. Images were captured at 1024 lines per image with a scan speed of 0.4 Hz, and 50 × 50 μm scans were acquired.

##### Optical Profilometry

Film thickness and surface roughness measurements were conducted using the Filmetrics Profilm3D Optical Profiler. Networks of nanosheets deposited on glass slides, were scratched using a tweezers, to have regions of the top surface and the underlying substrate visible in the FOV of the 50× objective lens. The interferometer was operated in white light interferometry mode, which uses a broadband source to generate interference patterns as the light returned from the surface recombined with light from a reference surface. The height at each pixel was stitched into a 3D reconstruction of the surface, which was leveled using the ProfilmOnline software. Step heights were determined using the histogram function, which plots each pixel height value, giving a bimodal distribution, with the difference between the peaks in the distribution giving the thickness of the network.

##### SEM, FIB‐SEM nt, and Image Analysis

Surface SEM and cross‐sectional SEM was images using a Zeiss Aruiga dual beam Ga FIB‐SEM at an accelerating voltage of 2 kV with an aperture of 30 μm. FIB‐SEM nt was carried out using the ZEISS ATLAS 5 software (Version 5.3.3.31) software. FIB‐SEM nt was carried out by depositing a 20 × 15 μm tungsten pad on the sample surface, lines were etched in the pad in a specific geometry, the lines were then filled with platinum and more tungsten was deposited on top. The lines etched into the pad are divided into two categories, two lines starting on the outer edges of the pad moving toward the center which are used to measure the thickness between slices and three central lines which are used for correcting beam focus and stigmation during the run. Slicing was done using a 600 pA beam. The images were aligned in dragonfly. Brightness and contrast of the images was altered in Fiji^[^ ^75^ ^]^ and the images were then segmented into substrate, nanosheets, pores, and surface using the Trainable WEKA segmentation^[^ ^59^ ^]^ plugin in Fiji.^[^ ^75^ ^]^ Porosity and pore shape and size were measured in Fiji,^[^ ^75^ ^]^ the tortuosity factors were measured using Taufactor.^[^ ^60^ ^]^ Speckle noise was removed from the image using the despeckle function in Fiji,^[^ ^75^ ^]^ the surface roughness and surface angles were then calculated in MATLAB (see Supporting Information).

##### Room‐Temperature Electrical Measurements

All electrical measurements were carried out using gold electrodes (≈100 nm) thick deposited on top of the networks using a gold evaporator (FC‐2000 Temescal Evaporator). The electrode geometry was defined by a 50 μm thick stainless‐steel mask. For the transistor measurements five IDEs with a channel length of 50 μm and a channel width of 19.55 mm were deposited. For the EIS measurements IDEs with channel length of 50, 80, 100, 150, and 200 μm with a constant channel width of 19.55 mm were deposited. IV curves were measured using a Keithly 2400 source meter. Transistor measurements were carried out by drop casting 1‐ethyl‐3‐methylimidazolium bis(trifluoromethylsulfonyl)imide (EMIM TFSI, Sigma‐Aldrich) onto the network, the IL was then dried by leaving for 12 h under vacuum at a temperature of 70 °C. The sample was returned to room temperature and pressure, and three probe measurements were carried out using a Keithley 2612A source meter, connected to a Janis probe station. Measurements were carried out with a gate voltage sweep from −3 to 3 V with a source‐drain voltage of 1 V at a scan rate of 50 mV s^−1^. EIS was carried out using a Keysight E4990E, with a test fixture (16047E) and spring‐loaded gold probes to contact the sample. Measurements were carried out in potentiostatic mode at a voltage of 50 mV between 20 Hz and 30 MHz at a precision speed of 3.

##### Temperature‐Dependent Electrical Measurements

The low frequency real component of the sample impedance was measured from a frequency of 10 to 1000 Hz in the temperature range 20 °C to −120 °C using a broadband α High‐Resolution Impedance Analyzer (Novocontrol GmbH, Germany). The low frequency real impedance is expected to be identical to the DC resistance in this sample. The samples were placed inside a sample holder with a fitted Pt 100 Ω resistance temperature sensor in contact with the electrodes. The temperature of the sample was controlled inside a double wall cryostat and maintained by a heated N_2_ jet produced by evaporating liquid nitrogen inside a 50 L dewar (Apollo 50 by Messer Griesheim GmbH). The Quatro temperature controller controls the power supplied to the dewar and gas heater. The AC measuring voltage applied to the sample was set at 0.5 V.

##### Statistical Analysis

The data in the manuscript are presented as means ± standard error (SE) in the mean. Combined uncertainty, where applicable for FIB‐SEM nt analysis, was calculated as the root sum of squares of standard errors in the mean and segmentation error. A sample size of *n *= 678 network cross‐sections were used in the 3D network reconstructions and *n* = 170 surface tiles were used to calculate the surface angle heatmaps. No data were excluded from the analyses and the experiments were not randomized.

## Supporting Information

Supporting Information is available from the Wiley Online Library or from the author.

## Conflict of Interest

The authors declare no conflict of interest.

## Supporting information

Supplementary Material

## Data Availability

The data that support the findings of this study are available in the supplementary material of this article.
