# Supplementary material for: The Electrical and Morphological Characteristics of Networks of Mechanically Exfoliated Nanosheets
Source: Small Sci. 2025 Oct 21;5(12):e202500417. doi: 10.1002/smsc.202500417 (PMC12697857; doi:10.1002/smsc.202500417)
Supplement: Supplementary file 1 — Supplementary Material [file SMSC-5-e202500417-s001.pdf]

Supplementary information: The Electrical and Morphological Characteristics of Networks of Mechanically Exfoliated Nanosheets

Luke Doolan[1], Yigit Sozen[2], Eoin Caffrey[1], Emmet Coleman[1], Tian Carey[1], Anthony Dawson[1], Cian Gabbett[1], Oran Cassidy[1], Jagdish K. Vij[3], Zdeněk Sofer[4], Andres Castellanos-Gomez[2] and Jonathan N. Coleman[1]*

[1]*School of Physics, CRANN & AMBER Research Centres, Trinity College Dublin, Dublin 2, Ireland*

[2]*Instituto de Ciencia de Materiales de Madrid (ICMM-CSIC) Madrid E-28049, Spain*

[3]*Department of Electronic & Electrical Engineering, Trinity College Dublin 2, Dublin 2, Ireland*

[4]*Department of Inorganic Chemistry, University of Chemistry and Technology Prague, Technická 5,Prague 6, 166 28, Czech Republic*

*colemaj@tcd.ie (Jonathan N. Coleman)

Table of Contents

Section 1: Relationship between nanosheet length and width 2
Section 2: Pore shape and size analysis 2
Section 3: Surface planes for nanosheet alignment measurement 4
Section 4: Contact resistance from DC measurements 5
Section 5: Estimating volumetric capacitance and calculating mobility 6
Section 6: Impedance fitting equations 7
Section 7: Calculated nanosheet-junction values as a function of channel length 8
Section 8: Activation energy analysis for conduction mechanism determination 9
Biblography 10

Section 1: Relationship between nanosheet length and width

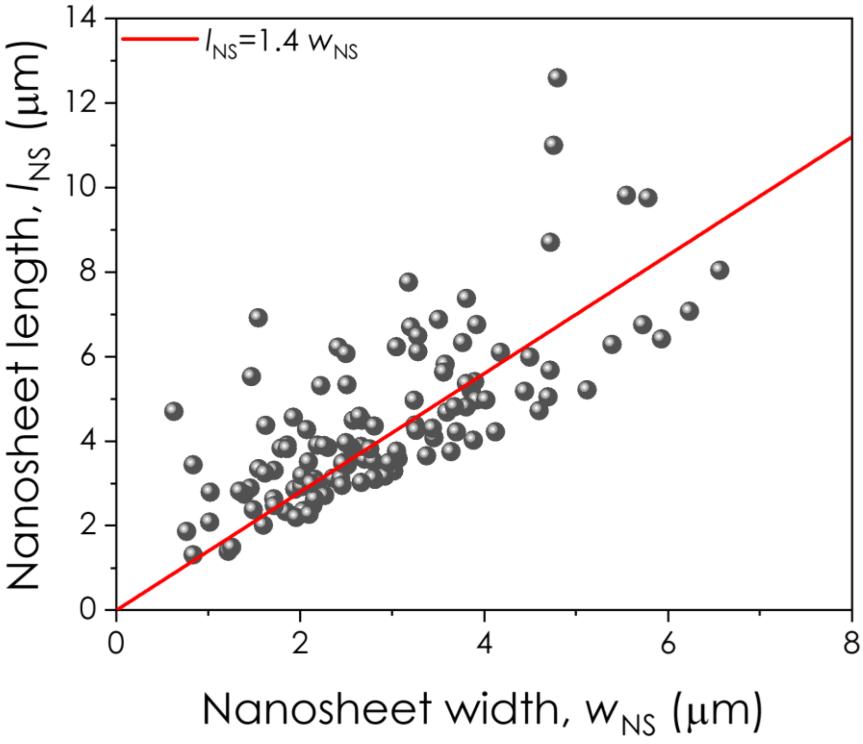

Supplementary figure 1-Nanosheet length vs width: Relationship between nanosheet length (l_{NS}, the longest direction) and nanosheet width (w_{NS}, length perpendicular to l_{NS}). The line is l_{NS}=1.4w_{NS}.

Section 2: Pore shape and size analysis

Due to the high degree of connectivity of the pore chambers within the pore network the pore shape and size must be calculated in 2D, we therefore calculate the pore size and circularity in each individual cross-sectional image, see ref.[1] for more detail. To understand the relative

importance of each measured pore we first plot the pore area and circularity as a 3D histogram and use the area-weighted pore fraction, f_a, which is given by

$$f_a = \frac{N_{Bin} A_{Bin}}{\sum_{All\,bins} N_{Bin} A_{Bin}} \qquad \text{(S1)}$$

Where N_{Bin} Is the number of pores in the bin and A_{Bin} is the average area the bin corresponds to.[1] The resulting data is plotted as a heat map in Figure SF2.

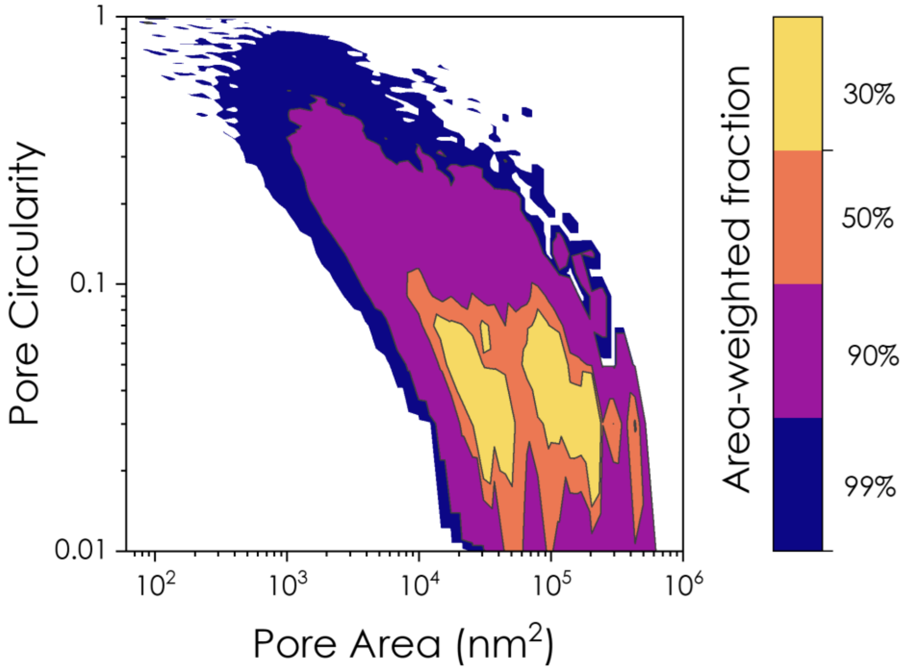

Supplementary figure 2- Pores shape analysis. Area-weighted pore fraction heat maps showing the relationship between pore area and shape as a function of the total porosity contained within the pores. The area weighted fraction was calculated using equation S1.

From the area-weighted pore fraction data we can calculate the average pore area and circularity by taking the average value of the bins which contain 90% of the total network porosity.

Section 3: Surface planes for nanosheet alignment measurement

To quantify the alignment of the nanosheets, planes were fit to the surface of the network reconstructions generated using FIB-SEM nt and the orientation of each was measured using MATLAB. A full description of this method can be found be found in ref.[2]. Briefly, the network surface height at each *x*,*z* point in the 3D image (Figure 2C, main text) was calculated by subtracting the maximum pixel value classified as substrate from the maximum pixel value classified as nanosheet in the *y*-direction (perpendicular to the substrate). The result is a surface height map where the value at each *x*,*z* coordinate is the height difference between the top of the nanosheet network and the substrate in the out-of-plane direction of the network (*y*-direction). A surface height map for the MPME MoS_2 network is shown in Figure 2E of the main text. To create the surface angle map (Fig. 2F, main text), the network surface was extracted as described above. This surface was then divided into a grid of equally sized tiles in the *x*,*z* plane, where the tile size can be varied on demand. Least squares fitting was then used to fit a plane to each tile in MATLAB, which describes the average orientation of the nanosheets at the network surface within each tile. This allows the polar angle between the normal vector to each plane and the *Y*-axis (the axis perpendicular to the substrate) to be calculated. A tile with a surface angle of 0° has a normal vector that is parallel to the *Y*-axis and lies perfectly flat in the plane of the film. Surface angles >0° suggest that there is disorder and misalignment of the nanosheets at the network surface.

The size of the plane fit to the surface can be varied, Figure S3A shows the measured average surface angle as a function of plane size. At very low plane sizes the calculated surface angle is more dependent on small fluctuations on the surface and is therefore not representative of the alignment of the nanosheets in the network. As the plane size increases the calculated surface angle decreases and converges on a value of ~2º. We use a plane size of 1100 nm, ¼ of the nanosheet length in the main text, shown as a red star in Figure S3A, as this angle should be representative of the average angle of a nanosheet. Figure 2F in the main text shows a heat map of the surface angles in the network. Figure S3B shows the distribution of nanosheet angles, the distribution shows a peak at small surface angles with a rapid decay as the surface angle increases.

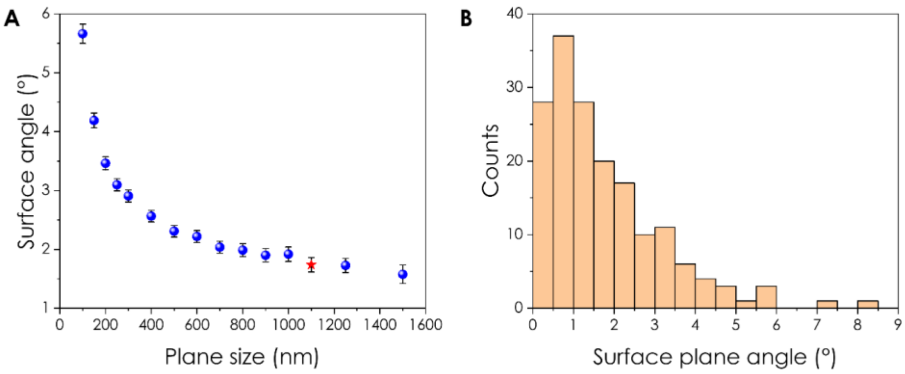

Supplementary figure 3- Surface angle analysis: A. Surface angle as a function of plane size. The data point at a plane size of 1100 nm, shown as a red star, was used for discussion in the main text. **B.** Distribution of surface angles calculated with a plane size of 1100 nm (*n* = 170).

Section 4: Contact resistance from DC measurements

In the main text contact resistance is calculated by a linear fit to the series resistance, calculated from impedance spectra, as a function of channel length. Traditionally, contact resistance is calculated from linear fits to DC resistance as a function of channel length, however, in nanosheet networks small variations in junction resistance in the channel can lead to large variations in the network resistance leading to incorrect and even negative values for the contact resistance. Figure S4 shows DC resistance as a function of channel length after the sample had been annealed at different temperatures, after some annealing steps negative contact resistances are calculated, highlighting the importance of using impedance measurements to calculate the contact resistance

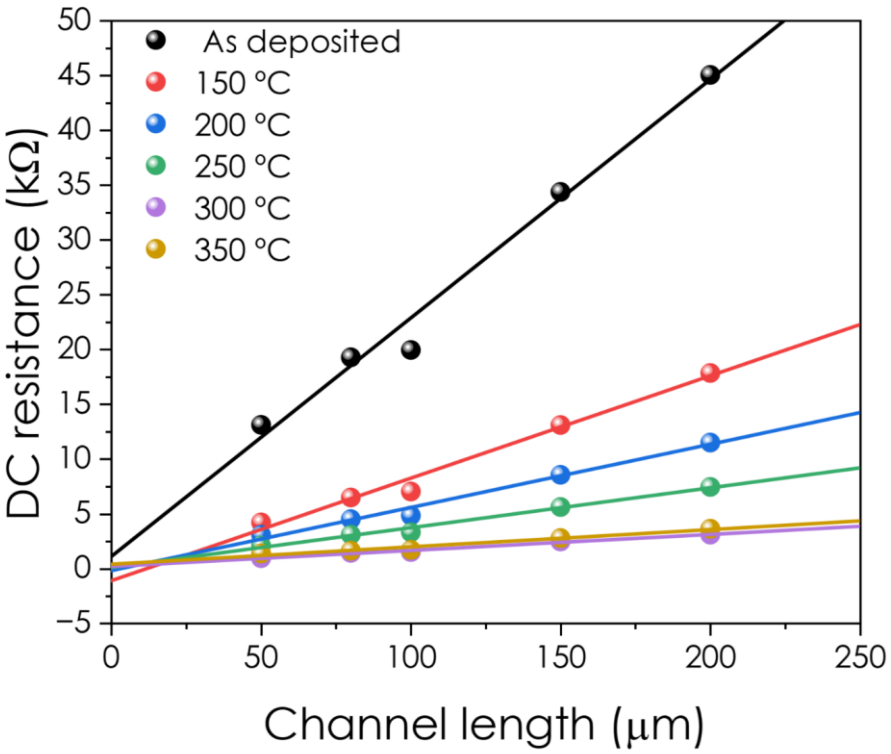

Supplementary figure S4: DC Resistance as a function of the channel length. Lines show linear fit to data, some lines show a negative y-intercept suggesting a negative value for the contact resistance.

Section 5: Estimating volumetric capacitance and calculating mobility

The areal capacitance (C_A) of a nanosheet network filled with ionic liquid will depend on various factors. For an EE MoS_2 network reported previously with a network thickness, $t_{NET,MOS2}$ = 25 nm and nanosheet thickness $t_{NS,MOS2} = 14\ nm$, we previously found C_A = 3.1 μF/cm^2.[3] However, C_A is dependent on both nanosheet thickness and network thickness because $C_A = C_V t_{Net}$ where C_V is the volumetric capacity of the network. This means the quoted value of C_A = 3.1 μF/cm^2 (3.1×10^{-2} F/m^2) [3] was consistent with a volumetric capacitance of C_V=1.24×10^6 F/m^3.

However, the volumetric capacitance depends on the nanosheet thickness (because this effects the nanosheet surface area). It has been shown that $C_V \propto 1/t_{NS}$.[4] Thus, the value of C_V=1.24×10^6 F/m^3 only applies to nanosheets with thickness $t_{NS,MOS2} = 14\ nm$, (see above).

However, using the scaling reported in the previous sentence, we can see that the volumetric capacitance of the nanosheets used here (2) can be related to the one given above (1) by: $C_{V2} = C_{V1} \times t_{NS1} / t_{NS2}$. Then, the volumetric capacitance of our nanosheets is

$$C_{V2} = 1.24 \times 10^6 F / m^3 \times 14 / 39 = 0.45 \times 10^6 F / m^3$$

Then, taking $\mu_{Net} = (dI_{ds} / dV_g) L_{Ch} / (C_V V_{ds} w_{Ch} t_{Net})$

And using:

dI_{ds}/dV_g=0.00385 S, channel length, L_{Ch}=50 μm, V_{ds}=1V, channel width, w_{Ch}=19.5mm, network thickness, t_{Net}=281 nm, yields a network mobility of 0.8 cm^2/Vs.

Section 6: Impedance fitting equations

The equation used to fit the real component of the network impedance spectra shown in Figure 3B in the main text is

$$Z'_{Net} = R_s + R_p \frac{(\omega R_p C_p)^n \cos\left(\frac{n\pi}{2}\right)}{1 + (\omega R_p C_p)^{2n} + 2(\omega R_p C_p)^n \cos\left(\frac{n\pi}{2}\right)} \quad \text{(S2)}$$

Where R_s and R_p are the series and parallel resistance, respectively, C_p is the parallel capacitance, *n* is the ideality parameter which is used to improve the quality of the fit in impedance spectroscopy and *ω* is the angular frequency of the applied voltage.

Similarly, the equation used to fit the imaginary component of the network impedance spectra is given by

$$Z''_{Net} = -\frac{R_p \sin\left(\frac{n\pi}{2}\right)(\omega R_P C_P)^n}{1 + (\omega R_P C_P)^{2n} + 2(\omega R_P C_P)^n \cos\left(\frac{n\pi}{2}\right)} \quad \text{(S3)}$$

Using equation 2 in the main text the network impedance spectra can be converted to the impedance of a nanosheet-junction pair. The real component of the nanosheet-junction resistance can be fit using

$$Z'_{NS-J} = R_{NS} + R_J \frac{(\omega R_J C_J)^n \cos\left(\frac{n\pi}{2}\right)}{1+(\omega R_J C_J)^{2n} + 2(\omega R_J C_J)^n \cos\left(\frac{n\pi}{2}\right)} \quad \text{(S4)}$$

Where R_{NS} and R_J are the nanosheet and junction resistance, respectively, C_J is the junction resistance and n is the ideality parameter, which describes the distribution of junction resistance and capacitance within the channel.

Similarly, the imaginary component of the nanosheet-junction impedance spectra can be fit using

$$Z''_{NS-J} = -\frac{R_J \sin\left(\frac{n\pi}{2}\right)(\omega R_J C_J)^n}{1+(\omega R_J C_J)^{2n} + 2(\omega R_J C_J)^n \cos\left(\frac{n\pi}{2}\right)} \quad \text{(S5)}$$

Section 7: Calculated nanosheet-junction values as a function of channel length

Using equation S4 and S5 the nanosheet and junction resistance, junction capacitance and ideality parameter can be calculated from fitting Z'_{NS-J} and Z''_{NS-J}. The calculated values as a function of channel length can be seen in Figure S5. In the main text the values calculated from the real component of the impedance spectra are discussed as these values have previously been found to be more accurate,[5] however, within the error of the measurement the values are all in agreement.

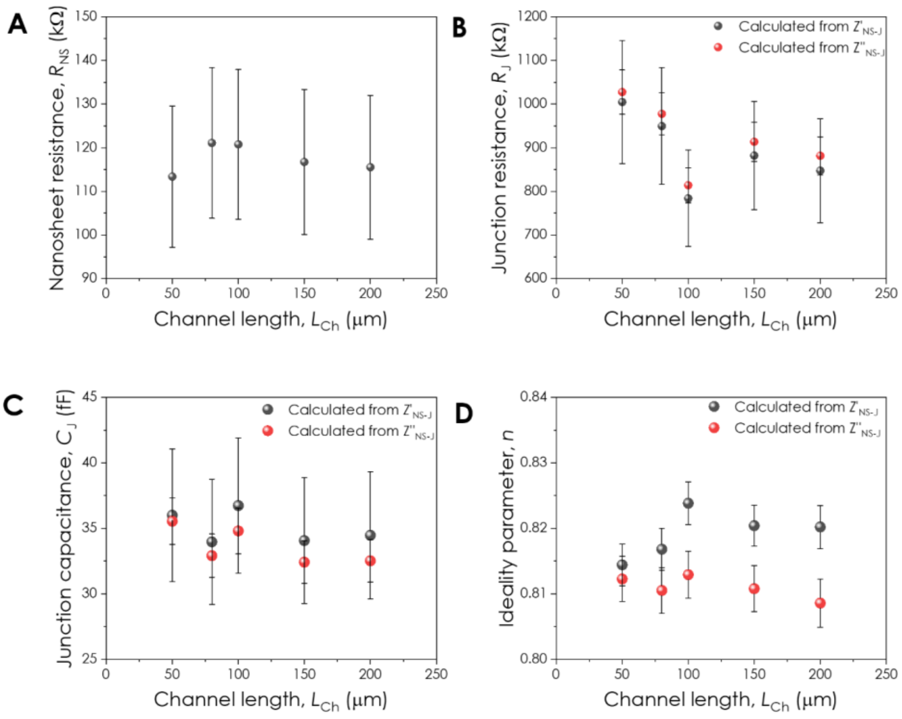

Supplementary figure S5: Nanosheet-junction values as a function of channel length. Calculated **A.** nanosheet resistance, **B.** junction resistance, **C.** junction capacitance and **D.** ideality parameter as a function of channel length. In B-D the values calculated from the real and imaginary impedance spectra are shown.

Section 8: Activation energy analysis for conduction mechanism determination

Determining the conduction mechanism from temperature dependent conductivity data can be difficult as a variety of different methods of conduction mechanisms often show reasonable agreement with the data. To overcome this problem, we employ activation energy analysis to determine the conduction mechanism. This method of determining the conduction mechanism is described in detail in ref.[6]. Briefly, first the reduced activation energy, W, is calculated

$$W = \frac{-T}{\rho}\frac{d\rho}{dT} \qquad \text{(S6)}$$

Where T is the temperature and ρ is the resistivity (the inverse of conductivity). From the slope of ln(W) vs ln(T) the conduction mechanism can be calculated. Here, we find the data is fit well by a line with a slope of −1 up to a temperature of 213 K, meaning the conduction mechanism is given by activated hopping. Below 213 K, the data shows an increasing slope, the reason for this is unclear.

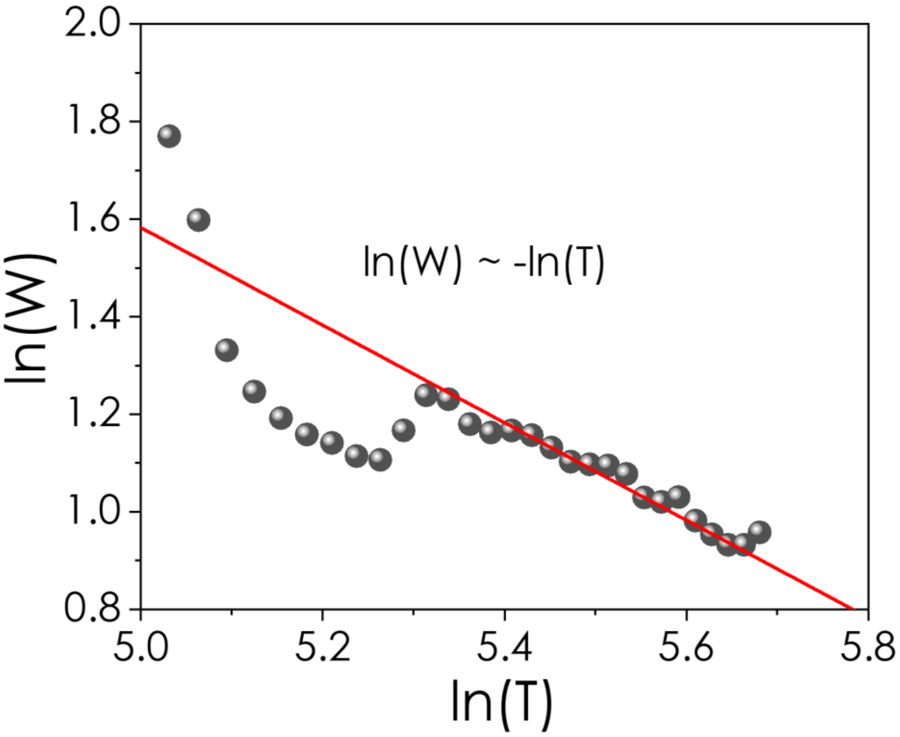

Supplementary figure S6: Reduced activation energy analysis. Reduced activation energy analysis for the temperature dependent conductivity data shown in Figure 3F in the main text. The fit of the line with a slope of 1 suggests the network shows activated hopping. The temperature mechanism appears to change at 213 K and the conduction mechanism below this temperature is not clear.

Biblography

1. Gabbett, C.; Doolan, L.; Synnatschke, K.; Gambini, L.; Coleman, E.; Kelly, A. G.; Liu, S.; Caffrey, E.; Munuera, J.; Murphy, C.; Sanvito, S.; Jones, L.; Coleman, J. N., Quantitative analysis of printed

nanostructured networks using high-resolution 3D FIB-SEM nanotomography. *Nature Communications* **2024,** *15* (1).
2. Caffrey, E.; Munuera, J. M.; Gabbett, C.; Doolan, L.; Neilson, J.; McCrystall, M.; McNamara, A.; Carey, T.; Coleman, J. N., Quantifying the influence of nanosheet aspect ratio on network morphology and junction resistance in solution-processed nanosheet networks. *Submitted* **2025**.
3. Carey, T.; Cassidy, O.; Synnatschke, K.; Caffrey, E.; Garcia, J.; Liu, S.; Kaur, H.; Kelly, A. G.; Munuera, J.; Gabbett, C.; O'Suilleabhain, D.; Coleman, J. N., High-Mobility Flexible Transistors with Low-Temperature Solution-Processed Tungsten Dichalcogenides. *ACS Nano* **2023,** *17* (3), 2912-2922.
4. Gholamvand, Z.; McAteer, D.; Harvey, A.; Backes, C.; Coleman, J. N., Electrochemical Applications of Two-Dimensional Nanosheets: The Effect of Nanosheet Length and Thickness. *Chem. Mater.* **2016,** *28* (8), 2641-2651.
5. Gabbett, C.; Kelly, A. G.; Coleman, E.; Doolan, L.; Carey, T.; Synnatschke, K.; Liu, S.; Dawson, A.; O'Suilleabhain, D.; Munuera, J.; Caffrey, E.; Boland, J. B.; Sofer, Z.; Ghosh, G.; Kinge, S.; Siebbeles, L. D. A.; Yadav, N.; Vij, J. K.; Aslam, M. A.; Matkovic, A.; Coleman, J. N., Understanding how junction resistances impact the conduction mechanism in nano-networks. *Nat Commun* **2024,** *15* (1), 4517.
6. Ippolito, S.; Urban, F.; Zheng, W.; Mazzarisi, O.; Valentini, C.; Kelly, A. G.; Gali, S. M.; Bonn, M.; Beljonne, D.; Corberi, F.; Coleman, J. N.; Wang, H. I.; Samori, P., Unveiling Charge-Transport Mechanisms in Electronic Devices Based on Defect-Engineered MoS2 Covalent Networks. *Adv Mater* **2023,** *35* (15).

Commented [CG1]: You could change the ref to Eoin's ACS nano paper from Submitted to Accepted here since it's at proofing stage?
